# Tumor immunomodulatory effects of polyphenols

**DOI:** 10.3389/fimmu.2022.1041138

**Published:** 2022-11-24

**Authors:** Qin Wang, Bin Yang, Nan Wang, Jian Gu

**Affiliations:** School of Pharmacy, Southwest Minzu University, Chengdu, Sichuan, China

**Keywords:** polyphenols, tumor immunity, natural compounds, bioavailability, MPNs

## Abstract

Polyphenols, commonly found in various plants, have attracted enormous attention due to their potential pharmacological activity, especially antitumor activity dependent on immune function. In recent years, the development of nanomedicine can counteract the low bioavailability of polyphenols and improve the effect of tumor treatment. Among them, metal-phenolic networks (MPNs), which utilize various metal ions and phenolic ligands for coordination binding, have now become candidates for polyphenol-based nanomedicine treatment of tumors. In this mini-review, we described the classification of polyphenols and their mechanisms in antitumor immune responses, and provided suggestions for the next steps of treating tumors with polyphenols.

## 1 Introduction

Natural polyphenols are one of the most widely distributed and abundant natural substances in nature (1). Recently, polyphenols have attracted extensive attention due to their extensive therapeutic effects in the anti-inflammatory, antioxidant and antitumor fields. Flavonoids, phenolic acids, lignans, and stilbenes are the four structurally major types of natural polyphenols (2). Among them, flavonoids are the most common one, accounting for about 60%, followed by phenolic acids accounting for about 30%. Numerous studies have shown that polyphenols have a variety of potential biological activities, especially antitumor activities due to the ability to modulate immune function (3).

The World Health Organization (WHO) estimates that tumors account for around 10 million death worldwide each year, placing them second only to ischemic heart disease in terms of mortality rates. The pathogenesis of tumors is characterized by changes in the ability of cells to proliferate, invade, and metastasize (4). Due to genomic instability, tumor cells have a high DNA mutations frequency. As a result, they can produce tumor antigens, which the immune system of the body identifies as non-self, and then trigger further immune responses. In recent decades, tumor immunotherapy has become the fourth tumor treatment besides surgery, chemotherapy, and radiotherapy, which has completely revolutionized tumor treatment (5). For example, for previously almost incurable metastatic melanoma, >50% of patients can now be relieved by combined anti-PD-1 and anti-CTLA-4 therapy (6).

With the development of nanotechnology, the potential of nanomedicine to improve the efficacy of tumor therapy has attracted interest. Among them, metal-phenolic networks (MPNs) have now become candidates for polyphenol-based nanomedicine treatment (7). The assembly of MPNs is mainly based on the cross-linking units between galloyl or catechol groups and metal ions. MPNs have the advantages of rapid preparation and pH responsiveness in the preparation process, and have received extensive attention in the field of nanomaterials for antitumor therapy.

Numerous studies have documented how polyphenols regulate the immune system. In this mini-review, we described the classification of polyphenols and the current understanding of the various mechanisms by which polyphenols could fight tumors by modulating the immune system, particularly the effects on adaptive and innate immune cells. Next, we describe the simple preparation process of MPNs *via* self-assembly and show the application in tumor immunotherapy, which may inspire further studies.

## 2 Classification of polyphenols

**Figure 1:**
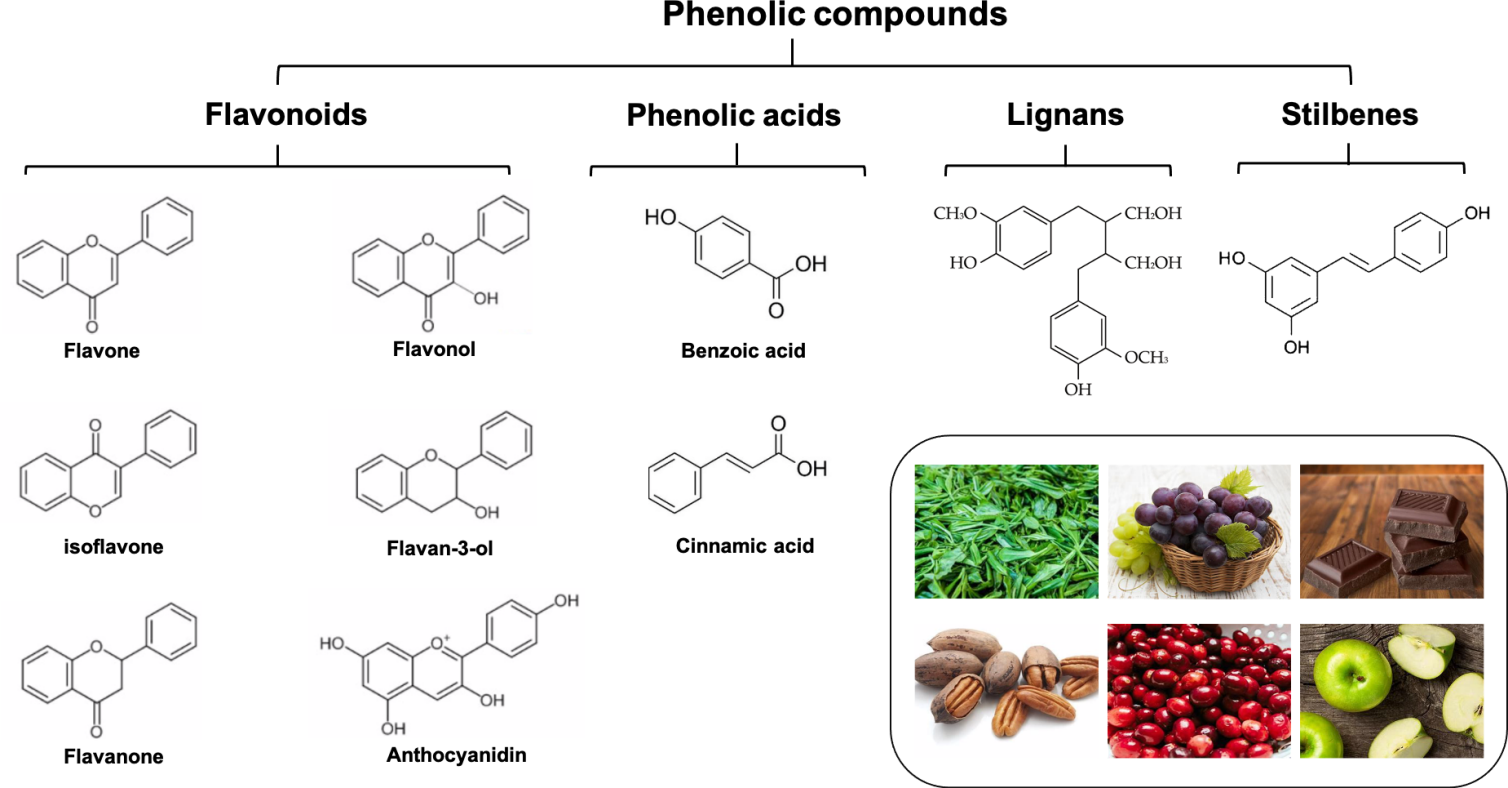
The basic classification of common polyphenols.

Natural polyphenols are the general term for polyhydroxy phenolic compounds. As one of the most widely distributed and abundant natural compounds in nature, they are widely found in various vegetables, fruits, grains, beans, tea, and other plants (1). Flavonoids, phenolic acids, lignans, and stilbenes are the four structurally major types of natural polyphenols (2). Among them, flavonoids are the most common one, accounting for about 60%, followed by phenolic acids accounting for about 30%. Generally, the term “flavonoids” refers to a group of C6-C3-C6 compounds made up of two benzene rings (the A-ring and the B-ring) joined together by a central three-carbon bond (C3). Glycosides are the primary form in which biologically active flavonoids are found in nature. The following categories of flavonoids can be distinguished based on the properties of the C3 structure’s oxidation levels and the B-ring’s position: flavonols, flavones, flavanones, flavan-3-ols, isoflavones, and anthocyanidins, etc. Phenolic acids are a class of compounds with at least one organic carboxylic acid on the same benzene ring. Common phenolic acids are caffeic acid, gallic acid, chlorogenic acid, ferulic acid, gallic acid, protocatechuic acid, etc. According to the different chemical structures, they are mainly divided into two categories: benzoic acid (C1–C6) and cinnamic acid (C3–C6) (8). Lignans are a class of natural substances formed by the polymerization of two phenylpropanoid monomers (C6-C3), usually in the dimer form, and sometimes in the trimer or tetramer form. Stilbenes are a general term for a class of substances with a 1-(2-Phenylethenyl) benzene or its polymer, which are found in low levels in normal tissues of the plant kingdom. **Figure 1** describes the basic classification of these phenolic compounds.

Natural polyphenols have a wide range of physiological activities, such as antioxidant, free radical scavenging, anti-ultraviolet, antibacterial, and antitumor effects, are widely used in medicine, pesticides, and food additives. Some polyphenols, such as (-)-epigallocatechin-3-gallate (EGCG), (-)-epigallocatechin (EGC), (-)-epicatechin-3-gallate (ECG), and (-)-epicatechin (EC) found in green and black tea, can inhibit the growth of detrimental bacteria such as Helicobacter pylori, Staphylococcus aureus, Escherichia coli, Listeria monocytogenes, and Pseudomonas aeruginosa, as well as hepatitis C virus, influenza, HIV, and Candida (9). ECG also could inhibit the invasion of non-small cell lung cancer (NSCLC) cells by inhibiting the expression of matrix metalloproteinase-2 (MMP-2) and urokinase-type plasminogen activator (uPA) (10). Common flavonoids like flavonols, flavones, flavanones, and anthocyanidins have been shown to play an important role in glucoregulation by regulating the relevant signal pathway, such as promoting β-cell proliferation, reducing apoptosis, increasing insulin secretion, and reducing insulin resistance and oxidative stress (11). The primary mechanism of action of polyphenols was initially thought to lie in their direct antioxidant effects. Nevertheless, these effects are currently no longer considered important actions *in vivo*, as these compounds do not reach high enough concentrations in most tissues to have a significant effect in scavenging free radicals. With the deepening of research, the role of polyphenols in immune regulation has gradually attracted people’s attention.

## 3 Immune response against tumor cells

**Figure 2:**
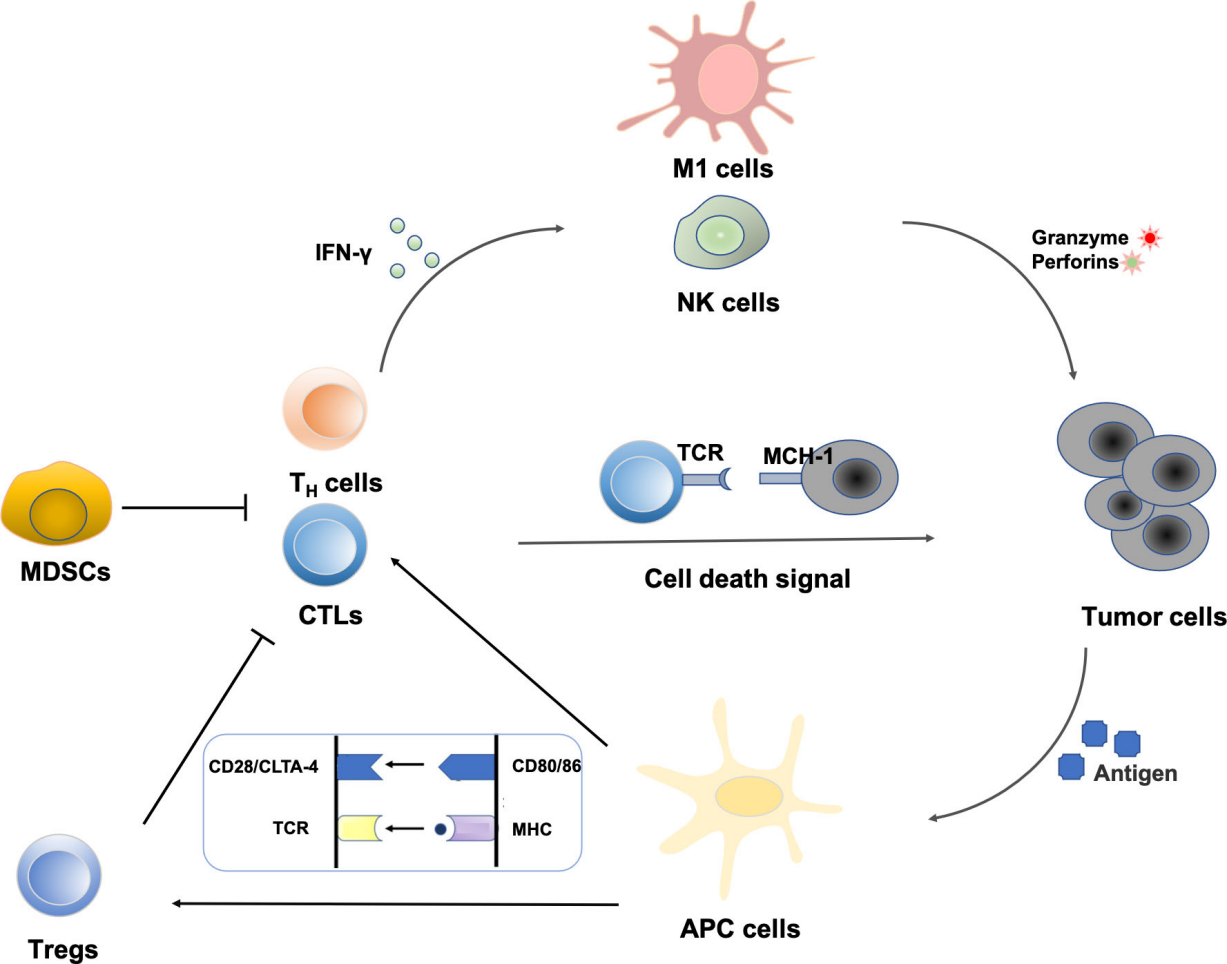
Regulation of immune cells in the tumor microenvironment: NK cells represent the first line of defense against a tumor cell. Immunogenic cell death increases the expression of costimulatory molecules (CD80/86) by APCs. Tumor peptide presentation to T cells leads to subsequent activation. CD8+ T cell-mediated elimination of tumor cells occurs *via* MHC-I recognition. Cytokines from T helper cells enhance the activity of macrophages and NK cells. MDSCs can also create the immunosuppressive cytokines that prevent CTLs and T_H_ cells from attacking tumors.

Polyphenols have a variety of biological activities and have great potential in immunomodulation. In recent years, immunotherapy has attracted great attention because of its remarkable efficacy for advanced tumors that are ineffective to conventional therapies, which holds promise for the treatment of advanced metastatic tumors (12, 13). Cancerous cells arise due to genetic mutations in oncogenes or suppressor genes. The high frequency of DNA mutations makes tumor cells usually carry a specific set of tumor-associated antigens (TAAs), which makes them easily recognized by immune cells *in vivo* and further triggers an immunogenic response (14). The identification of tumor antigens sparked research into the immune system’s relationship with malignancies and formed the “central dogma” of tumor immunotherapy. The innate immune system and the adaptive immune system are both parts of the immune system. Natural killer (NK) cells, macrophages, mast cells, monocytes, antigen-presenting cells (APCs), and other cells make up the innate immune system, which can inhibit tumor growth by triggering adaptive immune responses or directly killing tumor cells. The adaptive immune system functions mainly through T lymphocytes and B lymphocytes. T lymphocytes are transferred to the thymus through blood circulation and mature under the action of thymic hormones, while B lymphocytes mature and multiply in the bone marrow. T lymphocytes contribute to cellular immunity when triggered by antigens, and their immunological tasks are primarily to defend against intracellular infections, tumor cells, and heterologous cells. Meanwhile, B lymphocytes, stimulated by antigens, differentiate into plasma cells, and then secrete antibodies to participate in humoral immunity (15, 16). The regulating actions of significant immune cells in the tumor microenvironment are shown in **Figure 2**.

### 3.1 Role of macrophages

Macrophages are an essential component of the innate immune system of humans and are involved in tumor proliferation and migration. Macrophages are called tumor-associated macrophages (TAMs) in primary and secondary tumors, and they are the most numerous groups of cells in the tumor microenvironment, representing around 40% of the total number of cells. Macrophages are abundant in most tumor types and have complex functions in the tumor microenvironment (TME) (17). Macrophages can eliminate tumor cells by phagocytosis or induce tumor cell apoptosis by producing soluble factors. According to the different roles of macrophages in the tumor microenvironment and the types of activation of macrophages, TAM can be divided into M1 type and M2 type (18). At the early stage of tumorigenesis, TAMs exhibit the M1 phenotype, and as tumors progress, TAMs gradually polarize to the M2 phenotype. M1 macrophages, also known as “classically activated macrophages”, are activated by interferon-γ (IFN-γ) and LPS stimulation, which can highly express IL-23, IL-12, NO, and reactive oxygen species (ROS). It can eliminate tumor cells by phagocytosis and induce tumor cell apoptosis by producing soluble factors. M2 macrophages, also known as “alternatively activated macrophages”, are stimulated and activated by IL-4 and IL-13, which promote immunosuppression and angiogenesis by secreting various growth factors, chemokines, and cytokines, thereby driving tumor progression. In addition, studies have shown that TAM can also exert inhibitory functions by expressing a variety of receptors or ligands, such as expressing Arginase-1(Arg-1) to inhibit the activities of T cells, or by expressing PD-L1 and PD-L2 to promote the depletion of T cells and immune evasion of tumors (19).

### 3.2 Role of myeloid-derived suppressor cells (MDSCs)

Through a variety of methods, tumors might avoid immune detection. MDSCs are one such mechanism. Under the influence of chemokines released by tumors, immature myeloid cells and myeloid progenitor cells in the bone marrow give rise to MDSCs (20). Under normal circumstances, myeloid-derived precursor cells can rapidly differentiate into mature granulocytes, DCs and macrophages. Under pathological conditions, these myeloid-derived precursor cells are blocked from maturation and can become MDSCs with immunosuppressive function (21). MDSCs can deplete L-arginine, chelate L-cysteine or increase indoleamine-2,3-dioxygenase (IDO) activity by overexpressing Arg-1. This ultimately results in a deficiency of key trophic factors required for T cell proliferation (22). However, MDSCs can also create NO, ROS, and peroxynitrite (PNT), the latter of which prevents CD8+ T cells from attacking tumors by nitrating chemokines. Additionally, MDSCs are capable of secreting the immunosuppressive cytokines IL-10 and transforming growth factor-β (TGF-β), which promote the activation of regulatory T cells (Treg) and have an impact on macrophage polarization (23, 24). Further studies revealed that after MDSCs metastasized to the tumor, their exposure to the inflammatory and hypoxic tumor microenvironment clearly resulted in Arg1 and inducible nitric oxide synthase (iNOS) and decreased ROS production. At the same time, the chemokines CCL4 and CCL5 produced by MDSCs can induce Treg further into the tumor (25, 26). These conditions lead to stronger nonspecific immunosuppressive activity of MDSCs. In terms of tumor therapy strategies targeting MDSCs, we suggest that by blocking the production of MDSCs and promoting their maturation, the maturation of MDSCs may lead to the transformation of MDSCs from immunosuppressive cells to anti-tumor immune cells.

### 3.3 Role of natural killer (NK) cells

NK cells are effector cells that exert cytotoxic functions in the innate immune system (27). To keep track of malignant cells, a wide variety of activating and suppressing receptors can be displayed on the cell surface. NK cells and T lymphocytes, which are innate immune cells and acquired immune cells, respectively, are two types of cells with direct killing capability in the host. And they each have unique properties for identifying and eliminating tumor cells. Compared with T lymphocytes, NK cells have unique advantages in tumor immunotherapy: NK cells have a short survival time in the body, so the unpredictable risk is low (28). After activation, NK cells can release perforin and granzyme B to directly induce tumor cell death. Studies have shown that under the same conditions, NK cells have a stronger killing ability than CTL (29). Granzyme is a serine protease that cleaves intracellular proteins through caspase-independent or caspase-dependent pathways to induce apoptosis. Additionally, NK cells have a role in controlling T cell function. For example, IFN-γ excreted by NK cells can upregulate the sensitivity of CTLs, promote T cell immune responses and the formation of memory T cells. Regulatory T lymphocytes (Tregs) in the tumor have also been shown to be inhibited by IFN-γ, reversing the immunosuppressive condition concurrently (29). Depletion of NK cells may lead to extreme exhaustion of T cells, and even blockade of the immune checkpoint TIGIT does not significantly restore T cell function. However, tumor cells can also produce prostaglandin E2 to disrupt the function of NK cells, leading to immune escape (30). In addition, studies have shown that tumor immunotherapy based on allogeneic NK cells transplantation has not seen severe uncontrollable host versus graft reaction, indicating the feasibility of allogeneic NK cells therapy, and harnessing NK cells for tumor therapy is a promising option.

### 3.4 Role of T lymphocytes

As a key component of cellular immunity, T lymphocytes are crucial for both tumor immune evasion and antitumor immunity and have become a hot spot in tumor immunology research (31). A series of specific molecular markers (such as TCR, CD3, CD4, CD8, CD28, etc.) are present on the surface of T lymphocytes, among them, TCR is a molecule that specifically recognizes antigens and mediates immune responses on the surface of T lymphocytes (32, 33). Antigens will be produced during the occurrence and development of tumors, which will be captured and processed into immunogenic polypeptides by antigen-presenting cells (APCs), and exist on the surface of APCs in the form of antigen peptide-major histocompatibility complex (MHC) molecular complexes. Only when the antigen peptide-MHC molecular complexes bind to the TCR, and the co-stimulatory molecules expressed by APCs bind to the corresponding ligand on the surface of T lymphocytes, could the specific T cell immune response be activated. The costimulatory receptor on T lymphocytes that have received the most research is CD28. It interacts with the costimulatory receptors CD80 or CD86 (also known as B7-1 and B7-2, respectively) on the surface of APCs, which are essential for T lymphocyte activation. T lymphocytes are split into two significant subsets, the CD4+ T cells, and the CD8+ T cells, as a result of the specificity recognition of various types of MHCs (34, 35). CD4+ T cells primarily identify MHC class II and mediate adaptive immunity to a variety of tumor-related pathogens, among which T helper (TH)1 cell subsets play a critical role in antitumor effects. Additionally, 5% to 10% of the CD4+ T cells in healthy individuals’ peripheral blood are regulatory T (Treg) cells, an immunosuppressive subgroup of CD4+ T cells. Treg cells can block the actions of other T cells, macrophages, and NK cells by secreting immunosuppressive factors, and participate in tumor immune escape (36, 37). MHC class I-restricted CD8+ cytotoxic T lymphocytes (CTLs) could initiate tumor cell apoptosis by secreting cytotoxic granules containing perforin and granzyme, or indirectly kill cancer cells by secreting cytokines such as IFN-γ and tumor necrosis factor (TNF) (38). CTLs play a central role in tumor cell immunity, and their infiltration into tumor tissue is seen as a favorable prognostic factor in most tumors.

### 3.5 Tumor immune escape

As one of the important characteristics of tumorigenesis and development, tumor immune escape has a very complex mechanism, involving the participation of genes, metabolism, vascular production and other aspects (6, 39). Tumor cells can inhibit the activation of T cells by endocytosing antigens, or escape CTLs and NK cells-mediated lysis by overexpressing non-canonical MHC-I molecules, ultimately evading the immune system. At the same time, studies have found that immune checkpoints can act as negative regulators in the tumor microenvironment to mediate self-tolerance and promote tumor immune escape. Inhibitors targeting immune checkpoints (ICIs) have demonstrated good therapeutic efficacy in the treatment of a variety of solid tumors, becoming a landmark development in the history of treating cancer (40). Programmed death receptor-1 (PD-1) and programmed death ligand-1 (PD-L1) are the essential immune checkpoint molecules, their mechanism in tumor immune escape and the potential application in therapy are the hot spot of current tumor research (41). PD-1, also known as CD279, is commonly displayed on the surface of activating CD4+ T cells, CD8+ T cells, and DCs. PD-1 shares 30% homology with the cytotoxic T lymphocyte-associated antigen 4 (CTLA-4). The expression of PD-L1, which interacts with PD-1 on the membrane of T cells to prevent T cell activation and block tumor-killing actions, is upregulated on the membrane of tumor cells during carcinogenesis. As an immune checkpoint with similar effects to PD-1, CTLA-4 is also highly anticipated. In 1995, Krummel and Allison discovered that CTLA-4 is present on the surface of activated T cells, and its affinity for CD80 and CD86 ligands is much higher than that of CD28 (40). According to additional studies, tumor cells can activate CTLA-4, reduce the number of activated T cells, and prevent the formation of memory T cells, all of which contribute to the tumor immune escape (42). Since various tumor immune escape mechanisms are in a complex immune network, we can try to use comprehensive therapy targeting the combined action of multiple escape mechanisms.

The tumor microenvironment (TME) is a particularly complex ecosystem. In addition to the macrophages, MDSC, NK cells, and T cells mentioned above, other immune cells such as neutrophils, mast cells, and monocytes also play key roles in tumor immunotherapy. Interactions among these cell types can remodel the TME and regulate tumor progression. Combination therapy of multiple immune cells will be a strategy to improve anti-tumor efficiency in the future. In recent years, great progress has been made in understanding the regulation of these immune cells, especially the concept of the immune checkpoint, which enables the elucidation of new anti-tumor mechanisms that can be targeted by new therapeutic agents.

## 4 Immunomodulatory effects of polyphenols in the tumor microenvironment

**Figure 3:**
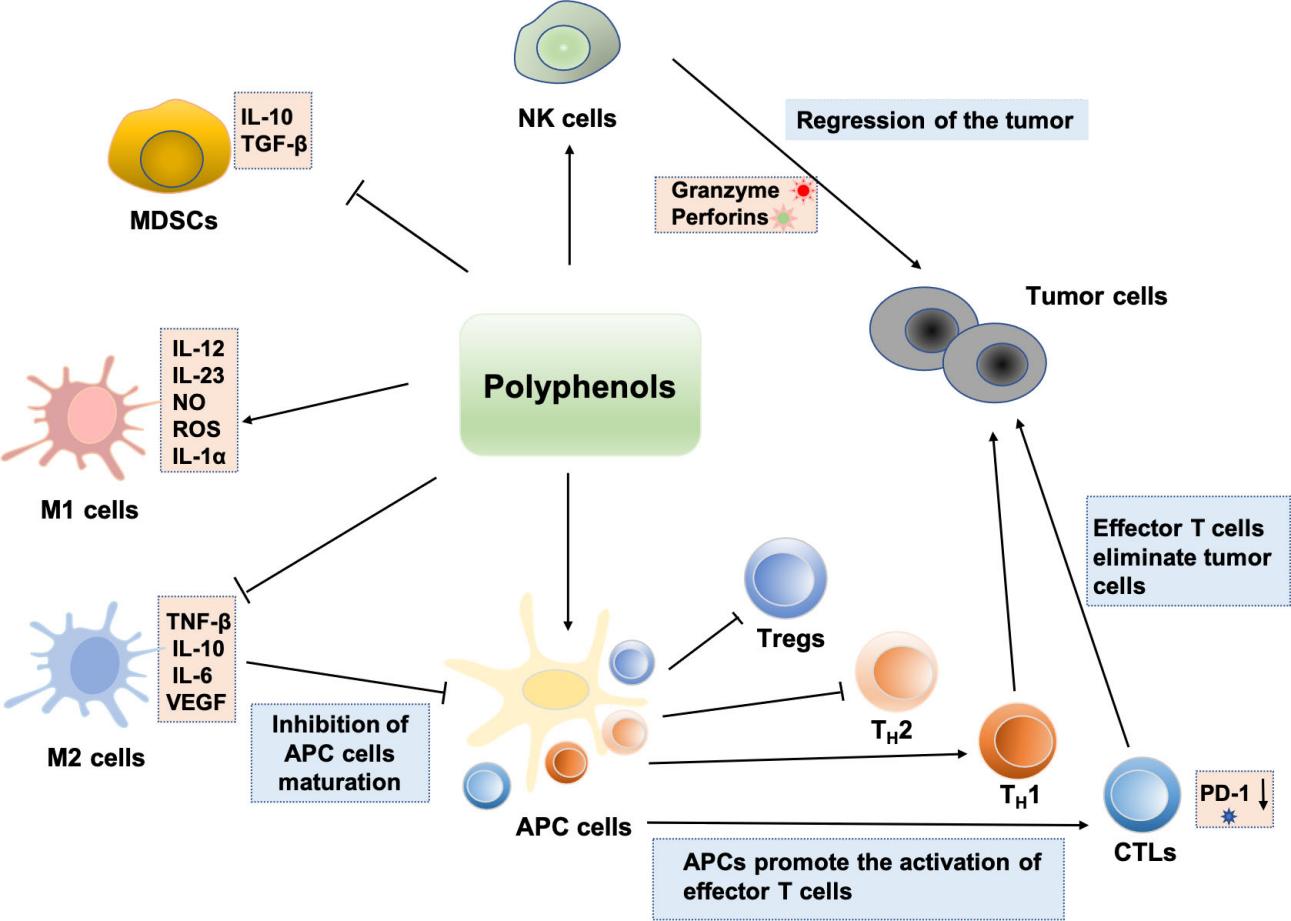
Regulation effects of polyphenols on immune cells: polyphenols can enhance NK cell antitumor activity. Some polyphenols can also induce immature MDSCs to differentiate into other mature immune cells. Polyphenols can inhibit the M2-like polarization of TAMs and enhancement the polarization of macrophages toward the M1 phenotype, which exerts antitumor effects. Polyphenols induce immunogenic cell death and induce cellular immunity by activating T_H_1 responses. Various polyphenols such as EGCG, genistein, and resveratrol can enhance the number and activities of CTLs. Bisdemethoxycurcumin decreases the level of PD-1 expression on the surface of CD8+ T lymphocytes, thus enhancing the CTLs’ cytotoxically activity and immune surveillance. In addition, Polyphenols induce tumor immunity by inhibiting the function of Treg and shifting from a T_H_2 or Treg balance to a T_H_1 response against the tumoral antigen.

Several biological activities of polyphenolic compounds have been described, and here we focus on the modulation of the immune system by polyphenols. The study found that polyphenols can block tumor immune escape and inhibit tumor growth by regulating immune cells in the tumor microenvironment, which offers a novel perspective on the investigation of polyphenols’ antitumor mechanism. The effects of polyphenols on a variety of significant immune cells are described in **Figure 3**.

### 4.1 Polyphenols and macrophages

The phagocytic activity of macrophages can be improved by polyphenols. Curcumin and epigallocatechin-3-gallate (EGCG) have been shown to trigger murine peritoneal macrophages and RAW 264.7 macrophages’ phagocytosis of fluorescent beads *in vitro* tests (43–45). In addition, polyphenols can control macrophage transcription of (inducible nitric oxide synthase) iNOS and their capacity to generate ROS. iNOS in macrophages can be inhibited by curcumin when lipopolysaccharide (LPS) is present, and rats fed a daily diet of curcumin (30 mg/kg) for two weeks saw a reduction in their capacity to produce ROS (46, 47). Additionally, it was discovered that quercetin may inhibit the stimulation of extracellular signal-regulated kinase (ERK) and mitogen-activated protein kinase (MAPK) by LPS, hence preventing the generation of IL-1β, IL-6, and TNF-α by macrophages (48). In addition, the study also found that polyphenols can inhibit the M2-like polarization of TAMs (49). Resveratrol not only suppressed the lung tumor cell-induced M2 polarization, but also inhibited the formation of new lymphatic vessels by inhibiting the production of IL-10 (50). Curcumin, EGCG, and resveratrol were added to liposomes by Mukhereej et al. to treat glioblastoma cells or TC-1 cells that had an HPV infection. In addition to the reduced tumor cell proliferation, it was discovered that macrophages moved from the M2 phenotype, which is indicated by the elevated affinity of arginase and IL-10, to the M1 phenotype, which is marked by elevated expression of iNOS and low appearance of IL-12 (51).

### 4.2 Polyphenols suppress the MDSCs

Polyphenols can affect myeloid-derived suppressor cells (MDSCs), an heterogeneous population of cells determined by myeloid origin, immature state, and ability to effectively inhibit T-cell responses (52). In addition to inhibiting the signaling of some key transcriptional pathways in MDSCs, some polyphenols can also induce immature MDSCs to differentiate into other mature immune cells. In 2013, Santilli et al. found that a standard tea polyphenol preparation (polyphenol E, PE) was able to reduce tumor-infiltrating MDSCs and inhibit tumor growth in a spontaneous neuroblastoma mice model (no effect in immunodeficient NOD/SCID mice) (53). *In vivo* experiments, PE (5 μg/mL) can inhibit tumor growth by acting on MDSCs and CTLs. *In vitro*, PE promoted the differentiation of MDSCs into more mature neutrophilic MDSCs by inducing granulocyte colony-stimulating factor (G-CSF) and reduced the infiltration of MDSCs into neuroblastoma. Using a breast cancer model, Forghani et al. found that silibinin reduced the content of MDSCs in the peripheral blood, spleen, and local tumor, thereby relieving the immunosuppressive state in tumor-bearing mice, and finally the tumor volume was greatly reduced (54). Curcumin has also been found to alleviate the immunosuppression induced by MDSCs. Tu et al. confirmed that curcumin can not only reduce the numbers of MDSCs in tumor-bearing mice, but also induce the maturation of MDSCs by suppressing the stimulation of NF-κB and STAT3 signaling in MDSCs (55). Liu et al. also confirmed that curcumin can also significantly increase CD8+ T cell cytotoxicity in the tumor microenvironment and induce apoptosis of MDSCs. At the same time, it can also down-regulate the expression of effector molecules such as Arg-1 and iNOS, further directly or indirectly alleviating the immunosuppressive effect mediated by MDSCs (56).

Normally, MDSCs promote the immunosuppressive effects of macrophages and DCs by interacting with other cells, which can protect the organism from excessive Inflammatory reactions. Since polyphenols can affect cell functions by regulating related signaling pathways in MDSCs and even participate in the inhibition of inflammatory processes, they may cause excessive inflammation in the organism, which makes the benefits of polyphenols in disease treatment seem controversial.

### 4.3 Polyphenols promote NK cells activation

Since NK cells are the initial line of resistance against tumor processes, studying the regulation of NK cells by polyphenols may be a new therapy. The quantities of polyphenols have a significant impact on how they regulate the cytotoxic activity of NK cells. Resveratrol, for instance, has a biphasic impact, boosting NK cells’ cytotoxicity at low concentrations (0.075 to 1.25 g/mL) while suppressing it at high doses (20 g/mL) (57–59). Espinoza et al. recently showed that low dosages (25-37 M) of resveratrol treatment of leukemia cells can boost leukemia cell lines’ responsiveness to NK cells and induce the transcription of NKG2D in NK cells. However, resveratrol could inhibit NK activity at a high concentration (60 μM) (60). In contrast, the soy isoflavone and genistein could induce the expression of protease inhibitor 9 (PI-9) at very low doses (10 nM), which subsequently blocks the ability of NK. At high concentrations, it enhances the capability of NK cells to kill breast malignant cells (61, 62). The molecular mechanisms by which polyphenols promote NK cell activation involve different signaling pathways. For instance, c-Jun-N-terminal kinase (JNK) and extracellular regulated kinase (ERK) can be activated by resveratrol, which has been demonstrated to be necessary for NK cells’ cytotoxicity (63, 64). Further evidence for this conclusion was provided by the suppression of JNK and ERK by pharmacological blockers (SP600125 and PD98059) as well as the suppression of JNK-1 and ERK-2 by siRNA (57). Additionally, resveratrol can increase the expression of NKG2D, a MAP kinase pathway upstream signaling molecule that is crucial for NK cell-mediated malignant cells identification (64).

### 4.4 Polyphenols activate T cell-mediated immune responses

T lymphocytes are essential for the host’s cell-mediated immune response to malignancies, as was already mentioned. Therefore, the goal of contemporary tumor immunotherapy is to increase or trigger T-cell reactivity to specific antigens. Polyphenols have been found to modulate the function of several different T cell subsets (CTLs, T_H_ cells, and Tregs) to enhance immunological responses or avoid immune escape.

#### 4.4.1 Polyphenols and CTLs

Various polyphenols such as EGCG, genistein, and resveratrol can enhance the number and activities of CTLs (62, 64). Chen et al. found that resveratrol can up-regulate the expression of Th1 cytokines, mainly IFN-γ, and activate CD8+ T cells to increase tumor tissue infiltration. Meanwhile, the cytotoxicity of CD8+ T lymphocytes was further boosted by up-regulating the production of perforin, granzyme B, and FasL, which further altered the renal carcinoma microenvironment (35). Besides, EGCG could activate CTLs in a mice model, and subsequently prevented photo-carcinogenesis (64). Resveratrol and genistein could enhance the expression of IFN-γ in CTLs *in vivo* and *in vitro*, leading to immune stimulation (65).

#### 4.4.2 Polyphenols and T_H_ cells

Polyphenols could be involved in the direct regulation of T_H_ cells activation and polarization. Chen et al. revealed the therapeutic effect of resveratrol on renal tumors and found that resveratrol can change the phenotype of Th cells from Th2 lymphocyte expressing IL-4 and IL-10 to Th1lymphocyte secreting IFN-γ and TNF-β, further IFN-γ can activate macrophages and inhibit Th2 lymphocyte proliferation (66). At the same time, resveratrol and curcumin can also reduce the expression levels of CD28 and CD80 in CD4+ T cells, up-regulate the expression of CTLA-4, and regulate tumor growth (67). In addition, the protective effect of resveratrol was associated with TH cells, which reduced pro-inflammatory factors, Th1, and Th17 cells, possibly improving the immune recognition of human B-cell lymphoma (68).

#### 4.4.3 Polyphenols and Tregs

Studies have also shown that polyphenols can induce tumor immunity by inhibiting the function of Treg. Treg is a subgroup of T cells that use Foxp3 as a transcription factor to control immune reactivity *in vivo*. Min et al. studied that EGCG (10 mg/kg) could significantly inhibit T cell proliferation and reduce the fraction of CD4+Foxp3+ Treg cells, as well as inhibit the expression of indoleamine-2,3-dioxygenase (IDO) in CD11b+ DCs (69). In clinical treatment, after 2 weeks of curcumin treatment for lung tumor patients, the proportion of Th1 cells in the body of the patient increased, and the frequency of Tregs was reduced (70). Meanwhile, after 2 weeks of treatment for colon tumor patients, the suppression of Treg cells was observed, while the T effector cells (Foxp3^−^) increased (71).

### 4.5 Regulation of immune checkpoints by polyphenols

In recent years, studies have found that polyphenols can block tumor immune escape by affecting cellular immune checkpoints. *In vitro*, treatment of human melanoma cells with curcumin and apigenin could inhibit the expression of PD-L1 on the surface of the cell, suggesting that curcumin and apigenin may be alternative treatments for immune checkpoint blockade (72). In addition, in a bladder tumor model, treatment with bisdesmethoxycurcumin significantly decreased the level of PD-1 expression on the surface of CD8+ T lymphocytes, and simultaneously elevated IFN-γ and granzyme B release was detected, thus ultimately significantly inhibiting tumor development and enhancing survival. In addition, research has shown that Rhus verniciflua Stokes (RVS) can be used to treat cancer. 20 major compounds were isolated and identified from the EtOAc fraction of RVS, of which 4 different compounds (protocatechuic acid, caffeic acid, taxifolin, and butin) blocked the CTLA-4/CD80 interaction and could serve as potential immune checkpoint inhibitor blockers and may be used in Immuno-oncology therapy (73).

We have mentioned that the TME is a particularly complex ecosystem. In addition to the regulatory effects on macrophages, MDSCs, NK cells and T cells mentioned above, polyphenols also play a key role in other immune cells. To our knowledge, B cells, as the important part of adaptive immune system, also play an important role in tumor immunity. Polyphenols can affect tumor growth by regulating the function of B cells. Studies have shown that resveratrol and curcumin can significantly regulate B cells proliferation and the ability of antibody production (74). Through interaction with the immune cells, various polyphenols have a great potential to reduce the risk of tumors. Recently, the cancer immune response has received a lot of attention, and people are committed to finding therapies that can interact with the immune system to eliminate tumor cells. In this case, polyphenols may emerge as adjunctive therapies or even new therapeutic alternatives.

## 5 Bioavailability of polyphenols

The bioavailability of polyphenols is directly correlated with their biological characteristics. The maximum plasma concentration (Cmax) is typically used to evaluate bioavailability, which is defined by the U.S. Food and Drug Administration (FDA) as “the rate and extent of the active ingredient or active moiety is absorbed from a drug and becomes available at the site of action” (75). The release through plant matrices, absorption, transport, metabolism, and removal are some of the processes that contribute to the bioavailability of phenolic compounds from various sources (76). Therefore, a number of factors, including chemical structure, food habits, food manufacturing, and interactions with other food components, can affect the bioavailability of polyphenols. Additionally, significant host-related aspects include gastrointestinal absorption, plasma transport dispersion, polyphenol degradation, and elimination. In general, glycosylated polyphenols are absorbed in the gut more slowly than aglycone polyphenols (77). However, before being absorbed, polyphenols in the presence of glycosides or polymers go through a rapid metabolism in the small intestine. Before being absorbed, polyphenols like flavonols, isoflavones, and anthocyanins must first be digested by intestinal enzymes or colonic bacteria. Gallic acid and isoflavones are the most bioavailable polyphenols, followed by caffeic acid, flavanones, catechins, and quercetin glycosides in studies. Proanthocyanidins have the lowest bioavailability (75). These polyphenols are then subjected to further metabolism in the liver and small intestine, where they undergo methylation, sulfonation, or glycosylation. They then move into the bloodstream and reach their intended tissues. By the time they reach the plasma, the metabolites are already chemically distinct from the polyphenols found in eatable foods, and their biological properties may have changed considerably (78).

The use of numerous polyphenols or the conjunction of polyphenols with traditional cancer therapies can be employed for immunomodulation to address this flaw in the anti-tumor strategy. However, due to their low absorption, polyphenols can have negative effects on tumors by reducing the effective dose that is supplied (79, 80). Through in-depth research on oral bioavailability and the continuous emergence of new technologies in recent years, more and more chemical, pharmaceutical and biological methods have been used to improve the bioavailability of polyphenols. The advancement of nanotechnology in recent years has also offered a viable solution to these issues. The solubility, biodistribution, and stability of polyphenols can be improved by encasing them in nanoparticles. This can enhance the antitumor activity of these natural compounds, especially their immunomodulatory effects (81). However, the biodegradability, drug encapsulation efficiency, stimulus-responsiveness, and active targeting ability of nanoparticles containing polyphenols are not satisfactory. In order to accelerate the development and clinical transformation of polyphenol-containing nanoparticles, we should also further explore and utilize the properties and functions of materials, build a multifunctional polyphenol-based nanoplatform, and provide better anti-tumor strategies.

## 6 Application of MPNs in tumor immunotherapy

Nanomedicine, which aims to create new paths for disease diagnosis and therapy, has evolved quickly in recent decades. The pharmacokinetic characteristics of nanoparticle (NP)-based medications may differ from other conventional drugs due to their unique size, which is typically in the range of 10 to 200 nm (82). At the same time, due to the dysfunctional vasculatures, NPs will accumulate specifically in tumor sites, which is beneficial for tumor therapy (83, 84). Ongoing research on important phenol molecules shows their potential biomedical applications. Based on the universal bonding, reducing properties, and biocompatibility, polyphenols are also suitable for the production of long-term stable, multifunctional, biodegradable, and safe NPs. Hence, a large number of studies based on polyphenol NPs have been carried out to address many key problems in drug delivery, gene therapy, and multimodal biological imaging.

Metal-phenolic networks (MPNs), which make use of the coordination between phenolic ligands and metal ions, have become promising candidates in the field of nanomedicine. In the areas of chemical catalysis and biomedicine, metal ions are crucial. Polyphenols are compounds that are widely present in plants with potential health care effects. The mutual cross-linking of metal ions and polyphenol structures can play a synergistic effect. Abundant polyphenols and metal ions endow MPNs with multiple properties, broadening its application in tumor immune regulation. In 2013, Caruso’s group used iron ions (Fe^3+^) and natural polyphenol tannic acid (TA) to rapidly react on the substrate surface to form a multifunctional MPNs coating for the first time (85). Since then, the polyphenols mainly TA, ellagic acid (EA), EGCG, gallic acid (GA), anthocyanin (ACN), and various other flavonoids have been used to coordination with metal ions into thin films on solid surfaces (86, 87). The metal ions that form MPNs are mostly transition metal elements or lanthanide metal elements such as Fe^3+^, Al^3+^, Zn^2+^, Cu^2+^, and Ag^+^, etc (88). Among the commonly used phenolic ligands, TA is a food additive approved by the US FDA and occupies an important position in the family of natural polyphenolic compounds. Furthermore, in terms of assembly of MPNs, Caruso and co-workers employed a “one-step” assembly composed of Fe^3+^ and TA, and the deposition of MPN films on granular polystyrene templates occurred when organic ligands (TA) and inorganic cross-linkers (Fe^3+^) were mixed in water at ambient temperature (85). In addition to the “one-step” assembly, the “multi-step” routes through coordinated interactions are also the most commonly used procedures for preparing MPNs (89). The “multi-step” assembly has unique MPNs properties because the substrates are in separate solutions of excess phenolic ligands or metal ions. The formation of small Fe^3+^-TA networks in the -solution can be precisely controlled due to the removal of unabsorbed Fe^3+^ and TA during the incubation step of the “multi-step” assembly. The different assembly routes can greatly affect the coordination pattern and performance of the final MPN products.

Recently, a variety of multifunctional MPNs based on TA have been extensively explored, especially to promote an antitumor immune response. Based on the coordination of metal-phenol self-assembly for appropriate oxygen transport *in vivo* and X-ray-triggered ultrasensitive ROS production in the tumor microenvironment, Dai et al. developed a simple X-ray nano-processor (Hb@Hf-Ce6 NPs). It was shown in a lung metastasis model that the combination of RT-radiodynamic therapy (RDT) and PD-1 checkpoint blockade immunotherapy based on Hb@Hf-Ce6 NPs could dramatically enhance anti-tumor immune responses (90). A combination of semiconductor polymers encapsulated with ferroptosis inducer (Fe^3+^) and exosome inhibitor (GW4869) were used to create phototheranostic metal-phenolic networks for phototherapy (PFG MPNs), which could produce superior near-infrared II fluorescence/photoacoustic imaging detection accuracy for a precise photothermal therapy (PTT). The synergistic effect of PTT and antiexosome PD-L1 can improve anti-tumor immunity and boost the immunological memory towards lymph node metastases (91). Additionally, a doxorubicin-loaded tannin acid-iron network (TAF) coated with fibronectin (FN) was used by Xu et al. to create a versatile treatment nanoplatform for combined tumor chemotherapy/chemokinetic/immunotherapy. Ferroptosis in the tumor was significantly increased by iron-based chemodynamic treatment and DOX chemotherapy. The DOX-TAF@FN technology can directly target tumor cells with high overexpression of αvβ3 integrin through FN-mediated targeting. The efficacy of tumor therapy and related immune responses can also be further enhanced by further blocking immune checkpoints with PD-L1 antibody (92).

Although MPNs have received more and more attention, their development is still in its infancy, and their application can be more refined. With the continuous development of medical technology, combining targeting moieties such as polyethylene glycol, hyaluronic acid, folic acid, and antibodies with polyphenols to further construct MPNs to achieve high tumor selectivity will also become a research hot spot. MPNs, as a multifunctional nanoplatform, have broad prospects in biomedical fields such as drug delivery, bioimaging, and catalytic therapy, especially in tumor immunotherapy.

## 7 Conclusion

The arrival of modern immunotherapy as a new milestone in cancer treatment has led to a conceptual revolution in the management of many tumor types previously limited by a lack of treatment options. Especially in solid tumors, the majority of successes have been achieved with ICIs, and the scenario is growing rapidly. Anti-tumor drugs, including ICIs, are also actively being studied in combination with an ever-widening extensive of other agents. Polyphenols have been the focus of research owing to their multiple protective effects against cancer. Large amounts of clear evidence have indicated that polyphenols regulate the immune system, which in turn influences tumor formation and development. Nevertheless, polyphenols with various pharmacological effects may cause drug or food interactions when administered simultaneously with narrow therapeutic index drugs. Besides, polyphenols are poorly absorbed and biodistributed in humans, and have a higher metabolism and excretion, which may decrease the optimal dose given to cancerous cells. There are still many challenges for clinical application of polyphenols. While methods for increasing polyphenol bioavailability have improved over the past few decades, including combining various polyphenols with anticancer medications. Numerous *in vivo* and *in vitro* investigations have demonstrated that the mixtures suppress tumor growth more potently than individual polyphenolic substances. Furthermore, an effective method to boost the bioavailability of polyphenols, which can be delivered to target tissues in a regulated manner, is to create drug delivery systems based on nanotechnology. The MPNs, which utilize the coordination between phenolic ligands and metal ions, have become promising candidates for nanomedicine, especially through their services as multifunctional therapeutic nanoplatforms. MPNs have unique properties such as rapid preparation, pH responsiveness, and negligible cytotoxicity. In addition, MPNs can be further modified and functionalized to meet specific application requirements, aiming to reduce the off-target toxicity and enhance the effectiveness of cancer treatment. The nano-strategy may be the “key” to break down the barriers of polyphenols application. Therefore, the use of nano-strategies for the safe and efficient release of polyphenols is expected to further advance the field.

## Author contributions

QW wrote the manuscript. JG revised the manuscript. BY and NW performed the literature search and data analysis. All authors contributed to the article and approved the submitted version.

## Funding

The work was financially supported by the Natural Science Foundation of Sichuan Province (2022NSFSC1735) and the Fundamental Research Funds for the Central Universities (ZYN2022094).

## Conflict of interest

The authors declare that the research was conducted in the absence of any commercial or financial relationships that could be construed as a potential conflict of interest.

## Publisher’s note

All claims expressed in this article are solely those of the authors and do not necessarily represent those of their affiliated organizations, or those of the publisher, the editors and the reviewers. Any product that may be evaluated in this article, or claim that may be made by its manufacturer, is not guaranteed or endorsed by the publisher.
